# National video consultation service- changing the way we deliver future care

**DOI:** 10.1192/bjo.2021.468

**Published:** 2021-06-18

**Authors:** Alka Ahuja, Gemma Johns, Sara Khalil, Mike Ogonovsky

**Affiliations:** Aneurin Bevan University Health Board and TEC Cymru

## Abstract

**Aims:**

In March 2020, when the COVID-19 outbreak emerged, Technology Enabled Care (TEC) Cymru went into partnership with the Welsh Government and CWTCH Cymru to offer a safe solution to protect the NHS and the public by developing and rolling-out a National Video Consulting (VC) Service on an All-Wales basis.

The aim was to quickly develop and roll-out an NHS-approved communication platform (Attend Anywhere) to all primary, secondary and community care services, and into care homes, prisons, dentistry, optometry and pharmacy to offer video consultations to patients.

**Method:**

The NHS Wales Video Consulting (VC) Service used a robust mixed methodology of surveys and interviews with patients, families and professionals. The real-time quality improvement approach was invaluable to the team as findings continually informed the approach and direction.

**Result:**

Based upon 10,000 survey responses from patients and professionals, and more than 300 interviews the results demonstrate that video consulting is consistently high in satisfaction, clinical suitability and acceptability across a wide range of patient demographics and clinical specialties in Wales. The key findings are

Very high in patient and clinician satisfaction (slightly higher in patients).

Clinically suitable across a wide range of specialties, care sectors and Health Boards.

Very high in patient and clinician satisfaction (slightly higher in patients).

High acceptability of VC, which is believed to be associated to the ‘Welsh Way’ of digital implementation processes.

Consistent data patterns across patient demographics (age, gender, urban/rural location).

Consistent data patterns across clinical settings and Health Boards.

**Conclusion:**

There is large appetite for VC in Wales, with high potential of sustainability and long-term use beyond COVID-19. The service is now working with clinicians, patients, carers and policy makers to explore the long-term use and sustainability of video consultations in Wales

